# CD74 Signaling Links Inflammation to Intestinal Epithelial Cell Regeneration and Promotes Mucosal Healing

**DOI:** 10.1016/j.jcmgh.2020.01.009

**Published:** 2020-01-28

**Authors:** Laura Farr, Swagata Ghosh, Nona Jiang, Koji Watanabe, Mahmut Parlak, Richard Bucala, Shannon Moonah

**Affiliations:** 1 Department of Medicine, University of Virginia School of Medicine, Charlottesville, Virginia; 4 Department of Microbiology, Immunology, and Cancer Biology, University of Virginia School of Medicine, Charlottesville, Virginia; 2 Department of Medicine, Yale University, New Haven, Connecticut; 3 National Center for Global Health and Medicine, Tokyo, Japan

**Keywords:** Repair, IBD, MIF Receptor, Proliferation Pathways, Akt, protein kinase B, CD74, cluster of differentiation 74, DSS, dextran sodium sulfate, EphB, ephrin type B receptor, ERK, extracellular signal-regulated kinase, GST, Glutathione S-transferase, HBSS, Hank’s buffered salt solution, IBD, inflammatory bowel disease, MBP, maltose binding protein, MIF, macrophage migration inhibitory factor, PI3K, phosphatidylinositol-3-kinase, TEV, Tobacco Etch Virus, TNBS, trinitrobenzene sulfonic acid, TNF, tumor necrosis factor, WT, wild type

## Abstract

**Background & Aims:**

The inflammatory response to intestinal damage promotes healing through mechanisms that are incompletely understood. Gene expression of cluster of differentiation 74 (CD74), the receptor for cytokine macrophage migration inhibitory factor, is increased in patients with inflammatory bowel disease (IBD), however, the role of CD74 signaling in intestinal inflammation remains poorly understood. The aim of this study was to determine the functional role of CD74 signaling in intestinal inflammation.

**Methods:**

We studied the characteristics of CD74 protein expression in human IBD and experimental colitis. The functional role of CD74 signaling in the intestine was investigated using cellular models; wild-type, *CD74*^*-/-*^, and bone marrow chimera mice; neutralizing anti-CD74 antibodies; flow cytometry; immunohistochemistry; immunofluorescence; immunoblotting; and clustered regularly interspaced short palindromic repeats and associated protein 9 technology.

**Results:**

In IBD patients and experimental colitis, CD74-receptor protein expression was increased in inflamed intestinal tissue, prominently in the crypt epithelial cells. By using distinct but complementary chemical and non–chemically induced mouse models of colitis with genetic and antibody neutralization approaches, we found that CD74 signaling was necessary for gut repair. Mechanistically, we found that the macrophage migration inhibitory factor cytokine, which also is increased in colitis, stimulated the CD74 receptor, enhancing intestinal epithelial cell proliferation through activation of the protein kinase B and the extracellular signal-regulated kinase pathways. Our data also suggest that CD74 signaling in immune cells was not essential for mucosal healing.

**Conclusions:**

CD74 signaling is strongly activated during intestinal inflammation and protects the host by promoting epithelial cell regeneration, healing, and maintaining mucosal barrier integrity. Enhancing the CD74 pathway may represent a unique therapeutic strategy for promoting healing in IBD.

## Graphical abstract

**Figure:**
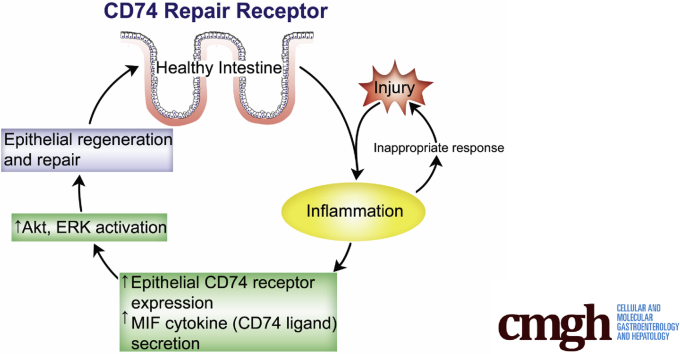

**Summary**

In this study, we uncovered a mechanistic link between intestinal inflammation and repair. We showed that cluster of differentiation 74 signaling is strongly activated during intestinal inflammation, and promotes mucosal healing by enhancing intestinal epithelial cell proliferation by activating the protein kinase B and extracellular signal-regulated kinase pathways.

Intestinal damage from colitis leads to significant morbidity worldwide. For example, inflammatory bowel disease (IBD) (exemplified by Crohn’s disease and ulcerative colitis), affects more than a million persons living in North America, and the global incidence of both disorders is increasing.^1^^,^^2^ This is compounded further by the fact that a significant number of patients with IBD either do not have a relevant response or relapse during treatment, and surgical resection of the inflamed colon (colectomy) is indicated when treatment of IBD has failed.^3^^,^^4^ Therefore, new therapeutic strategies are needed. Mucosal healing has emerged as an important treatment goal because it strongly predicts sustained clinical remission and resection-free survival in IBD.^4^^,^^5^ However, the mechanisms that promote the healing process are not fully understood. Further understanding of the mechanisms that promote healing could lead to new therapeutic opportunities for IBD patients.

Although excessive inflammation may extend injury, it is increasingly clear that the inflammatory response to intestinal damage is essential for repair.^6^^,^^7^ That said, the mechanisms that link inflammation to repair are incompletely understood. Cluster of differentiation 74 (CD74) is a membrane protein that initially was thought to function mainly as a major histocompatibility complex class II chaperone. Moreover, additional roles have been identified for CD74, such as serving as a receptor for the cytokine macrophage migration inhibitory factor (MIF).^8^^,^^9^ Increased gene expression of CD74 occurs in patients with IBD.^10^^,^^11^ However, the role of CD74 signaling in intestinal inflammation remains poorly understood. By using human studies, cellular models, and mouse models, we have uncovered a mechanism of intestinal healing during colitis involving CD74 signaling that promotes intestinal repair.

## Results

### CD74 Expression Is Increased in Patients With IBD

**Figure 1:**
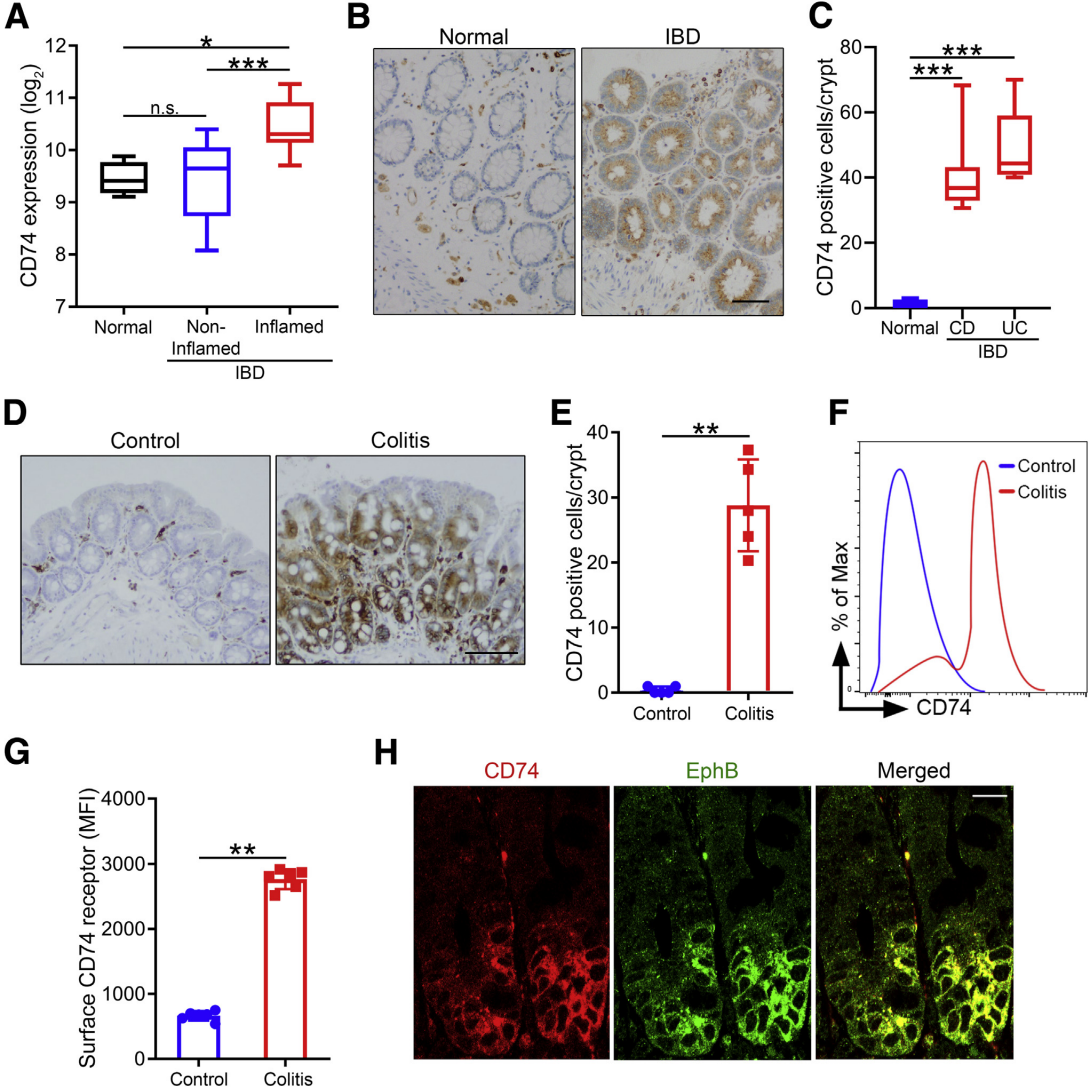
**CD74 expression is increased in IBD patients and experimental colitis.** (*A*) Increased CD74 expression in inflamed mucosa. CD74 gene expression levels in normal healthy controls, and in noninflamed and inflamed mucosa of IBD patients (n = 4, 16, and 12, respectively). *Bars* represent the medians. (*B* and *C*) Increased CD74 protein expression in epithelial cells of IBD patients. Immunohistochemical analysis of CD74 expression in mucosa of IBD patients (Crohn’s disease [CD], n = 8; ulcerative colitis [UC], n = 5) and healthy controls (n = 6). *Scale bar*: 50 μm. (*D* and *E*) Increased CD74 protein expression in epithelial cells in experimental colitis. Immunohistochemical analysis of intestinal CD74 expression in mice 24 hours after intracecal injection with 3% DSS in drinking water or normal drinking water control (n = 5 per group). *Scale bar*: 200 μm. (*F* and *G*) Increased surface CD74 expression on intestinal epithelial cells during colitis. Flow cytometry analysis of intestinal epithelial cells for CD74 surface expression (n = 6 per group). (*H*) Significant CD74 expression in crypt epithelial cells during colitis. Immunofluorescence analysis of intestinal tissue stained with anti-CD74 and EphB antibodies. *Scale bar*: 20 μm. One representative image of the 3 independent experiments performed is shown. Data represent means and SD. *P < .05, **P < .01, and ***P < .001. MFI, mean fluorescent intensity.

Given the potential importance of CD74 in IBD pathogenesis, we analyzed CD74 gene expression in inflamed and noninflamed colonic mucosa of IBD patients, and normal healthy controls. We found increased expression of CD74 in mucosa samples from inflamed areas compared with noninflamed and healthy control samples (Figure 1*A*). Also, no significant difference was found between noninflamed and healthy control samples (Figure 1*A*). To expand on these findings, we performed immunohistochemical analysis to characterize CD74 expression at the cellular level. Immunohistochemistry staining showed significantly higher expression of CD74 in colonic epithelial cells of IBD patients (Crohn's disease and ulcerative colitis) compared with healthy controls (Figure 1*B* and *C*).

Dextran sulfate sodium (DSS)-induced colitis is a commonly used mouse model that mimics key immunologic and histopathologic features of IBD in human beings.^12^^,^^13^ Consistent with our human findings, CD74 expression was enhanced in intestinal epithelium of mice with DSS-induced colitis as analyzed by flow cytometry and immunohistochemical staining (Figure 1*D*–*G*). In addition, increased CD74 expression was seen early, within 24 hours after DSS treatment. Notably, expression of CD74 protein was most intense in the crypt epithelial cells during inflammation (Figure 1*D*). Ephrin type B receptor (EphB) (EphB2/B3) protein is a marker of proliferating intestinal crypt cells.^14^ We found that CD74 co-localized with EphB in crypt epithelial cells (Figure 1*H*).

Taken together, these findings suggest that CD74 is up-regulated in intestinal inflammation, with a predominant expression pattern in the proliferating intestinal crypt cells.

### CD74 Promotes Mucosal Healing

**Figure 2:**
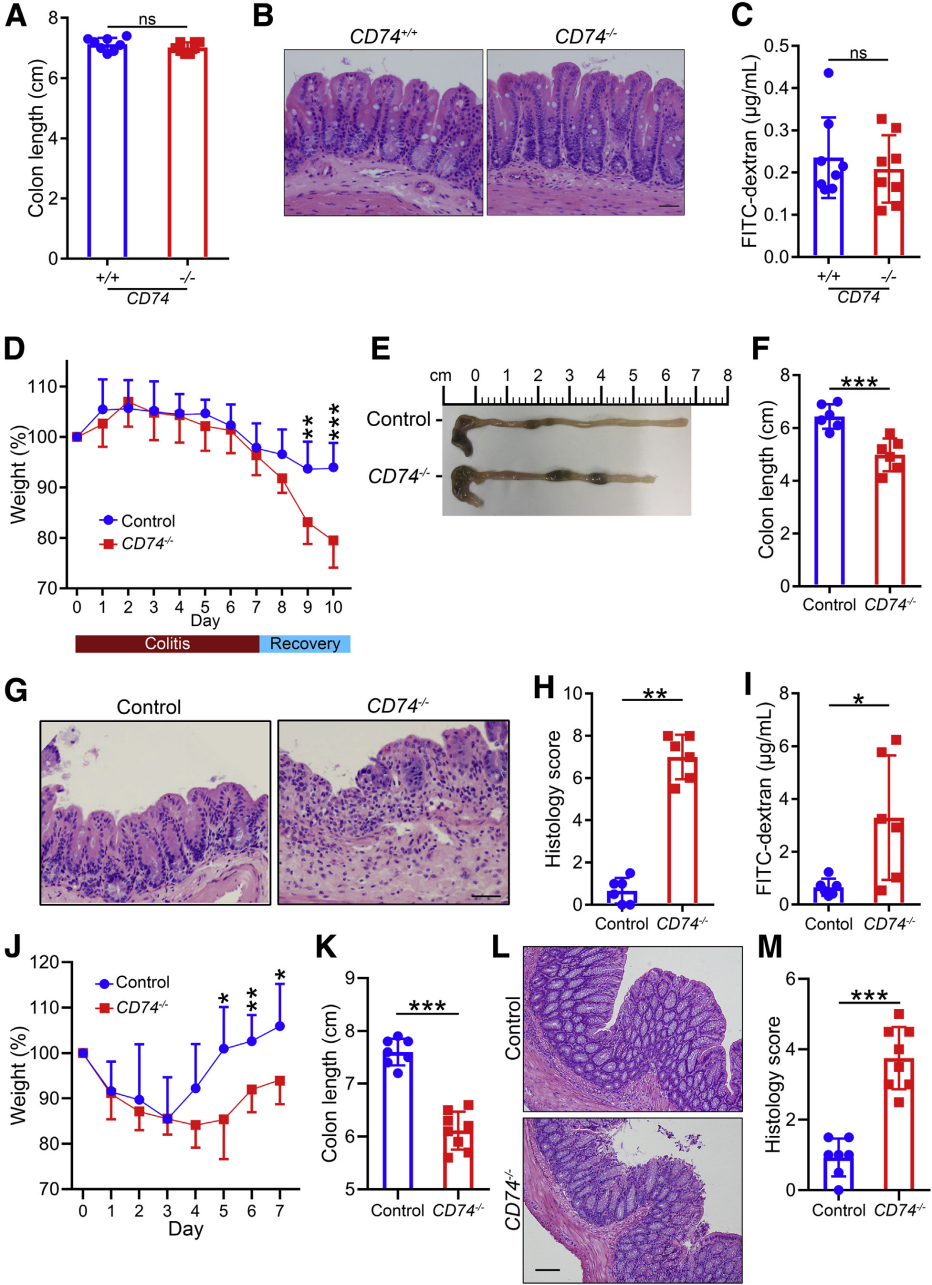
**CD74 promotes mucosal healing in DSS- and TNBS-induced colitis.** (*A*–*C*) CD74-deficient mice show normal intestine. Colon lengths, representative H&E-stained images, and fluorescein isothiocyanate (FITC)-dextran levels in WT (*CD74*^*+/+*^) and CD74-deficient mice (*CD74*^*–/–*^) are shown (n = 8 per group). (*D*–*I*) CD74 promotes repair. Body weight curves, colon lengths, H&E-stained images, and histology scores of WT control and *CD74*^*–/–*^ mice treated with DSS followed by a recovery period. Intestinal permeability measured by FITC-dextran in serum 4 hours after gavage (n = 6 per group). (*J*–*M*) Body weight curves, colon lengths, H&E-stained images, and histology scores of WT control and *CD74*^*–/–*^ mice after TNBS-induced chronic colitis (n = 7 and 8, respectively). *Scale bars*: 100 μm. Data represent means and SD. *P < .05, **P < .01, and ***P < .001.

Because proliferation of intestinal epithelial cells is a critical component of intestinal tissue regeneration and CD74 overexpression was most noticeable in proliferating crypt epithelial cells, we hypothesized that CD74 may have a direct role in intestinal tissue repair. To test the hypothesis that CD74 may function to promote recovery and mucosal healing in colitis, we treated mice with 7 days of DSS to induce inflammatory damage followed by a recovery period. Similar to wild-type (WT) mice, *CD74*^*−/−*^ mice showed normal colon, intestinal histology, and mucosal integrity, and lacked spontaneous colitis in the absence of pathologic insults (Figure 2*A–C*). During the colitis phase, WT and *CD74*^*−/−*^ mice showed comparable clinical disease, as evidenced by similar body weight loss (Figure 2*D*). However, as recovery commenced, WT mice began to show clinical improvement. On the other hand, *CD74*^*−/−*^ mice showed a course of progressive body weight loss and had to be euthanized because they met humane end point criteria (Figure 2*D*). In addition, *CD74*^*−/−*^ mice had significantly shorter colons compared with the WT mice (Figure 2*E* and *F*). Furthermore, *CD74*^*−/−*^ mice showed significant tissue damage with extensive epithelial loss and ulcerations on histopathologic examination (Figure 2*G* and *H*). Consistent with the severe ulceration observed in *CD74*^*−/−*^ mice, intestinal permeability was increased markedly in these animals, based on a serum fluorescein isothiocyanate–dextran analysis (Figure 2*I*). Given that CD74 was necessary for healing in DSS-induced colitis, we next used the 2,4,6-trinitrobenzene sulfonic acid (TNBS)-induced chronic colitis injury-repair model of human IBD^13^^,^^15^ as another disease model to evaluate its function. After the fifth cycle of TNBS, weight loss was similar between *CD74*^*−/−*^ mice and controls during the first 3 days. However, *CD74*^*−/−*^ mice had impaired weight gain during the recovery period (Figure 2*J*). In keeping with the delayed recovery from intestinal injury, *CD74*^*−/−*^ mice showed shorter colon lengths and greater epithelial loss on histopathologic analyses (Figure 2*K*–*M*). Thus, our data indicate that CD74 depletion leads to impaired healing in colitis.

### CD74 Is Necessary for Mucosal Healing in Amebic Colitis

**Figure 3:**
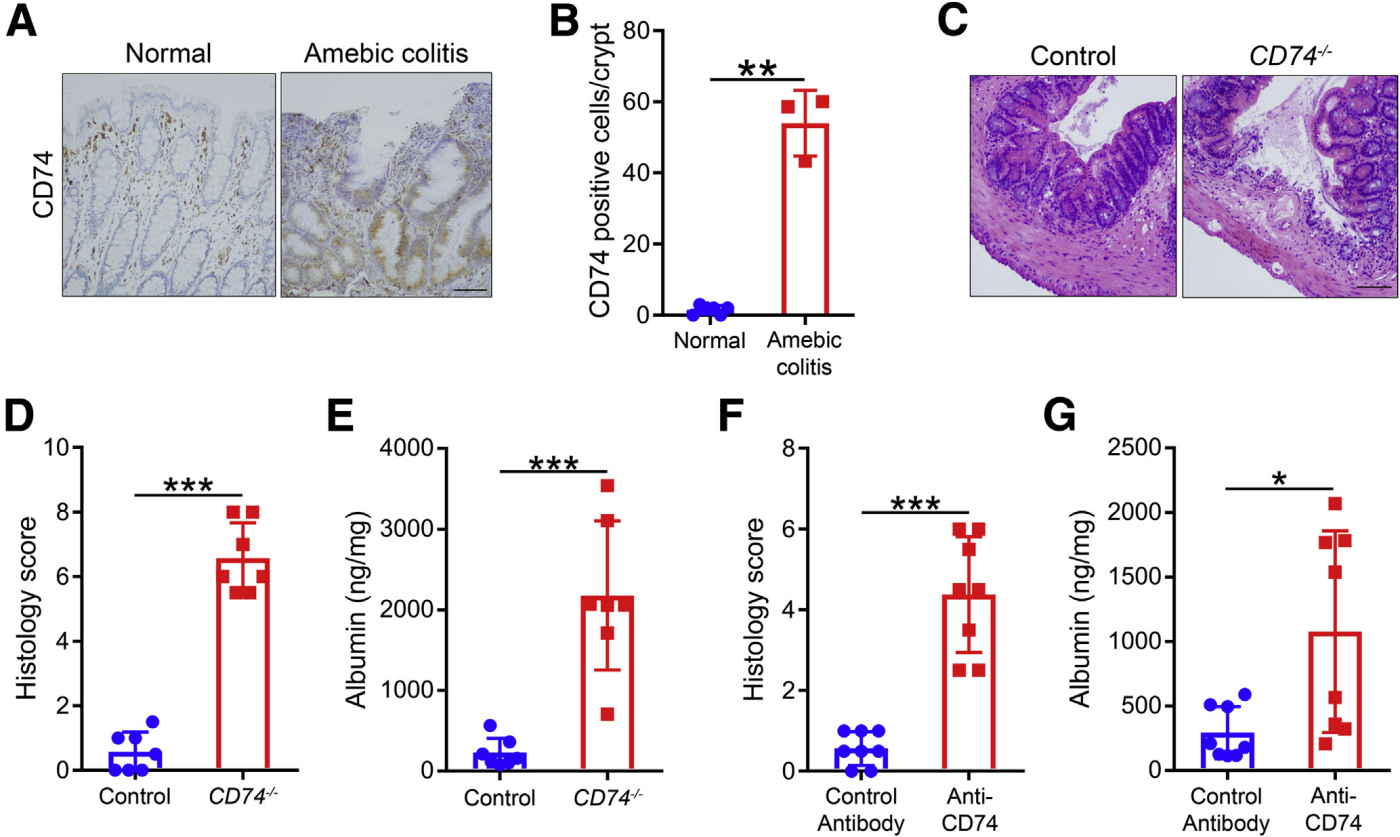
**CD74 promotes mucosal healing in amebic colitis.** (*A* and *B*) Increased CD74 protein expression in epithelial cells in human amebic colitis. Immunohistochemical analysis of CD74 expression in mucosa of patients with amebic colitis and normal healthy controls (n = 3 and 6, respectively). (*C*–*E*) H&E-stained images and histology scores of WT control and *CD74*^*–/–*^ mice after infection with *E histolytica*. Intestinal permeability measured by albumin levels in cecal luminal contents (n = 7 per group). (*F* and *G*) H&E-stained images, histology scores, and albumin levels in WT mice treated with anti-CD74 or control antibodies (n = 8 per group). *Scale bars*: 200 μm. Data represent means and SD. *P < .05, **P < .01, ***P < .001.

In a separate but complementary non–chemically induced mouse model of colitis, we took advantage of the amebic colitis and repair model to further examine the role of CD74 during intestinal inflammation and regeneration. Mucosal inflammation is a hallmark of amebic colitis, which is caused by the protozoan parasite *Entamoeba histolytica*.^16^, ^17^, ^18^, ^19^, ^20^, ^21^ Amebic colitis shares similar pathology to IBD, explaining why it often is misdiagnosed as IBD.^22^^,^^23^ In addition, similar to the IBD and chemically induced colitis, increased CD74 expression also was observed in amebic colitis (Figure 3*A* and *B*). During homeostasis, epithelial cells of the large intestine self-renew every 2–3 days.^24^ In B6 mice, amebic colitis is a self-limiting condition with initial inflammatory damage followed by infection clearance and recover within 72–96 hours.^25^ To further determine if *CD74*^–/–^ mice have compromised healing, we challenged mice with *E histolytica* pathogens. We found that *CD74*^–/–^ mice showed impaired healing from *E histolytica*–induced colitis, as evidenced by more severe histopathology, consistent with a defective ability to recover from intestinal injury (Figure 3*C* and *D*). Furthermore, *CD74*^–/–^ mice had significantly increased luminal albumin compared with WT mice, which also supports that *CD74*^–/–^ mice had impaired healing and consequently more severe barrier disruption (Figure 3*E*). In addition to a genetic approach, we performed inhibition of CD74 signaling in WT mice using neutralizing anti-CD74 antibody. Consistent with our findings in *CD74*^*−/−*^ mice, inhibition of CD74 signaling by blocking antibodies also resulted in more tissue damage (Figure 3*F* and *G*). There were no differences in parasite burden between WT and *CD74*^*−/−*^ mice. This also was true for groups given antibody control or anti-CD74 antibody, indicating that defective CD74 signaling did not alter the parasite infection rate and that groups were exposed to the same parasite levels. Therefore, CD74 is essential for mucosal healing in 3 distinct colitis mouse models.

### CD74 Stimulation Activates Proliferative Pathways in Intestinal Epithelial Cells

**Figure 4:**
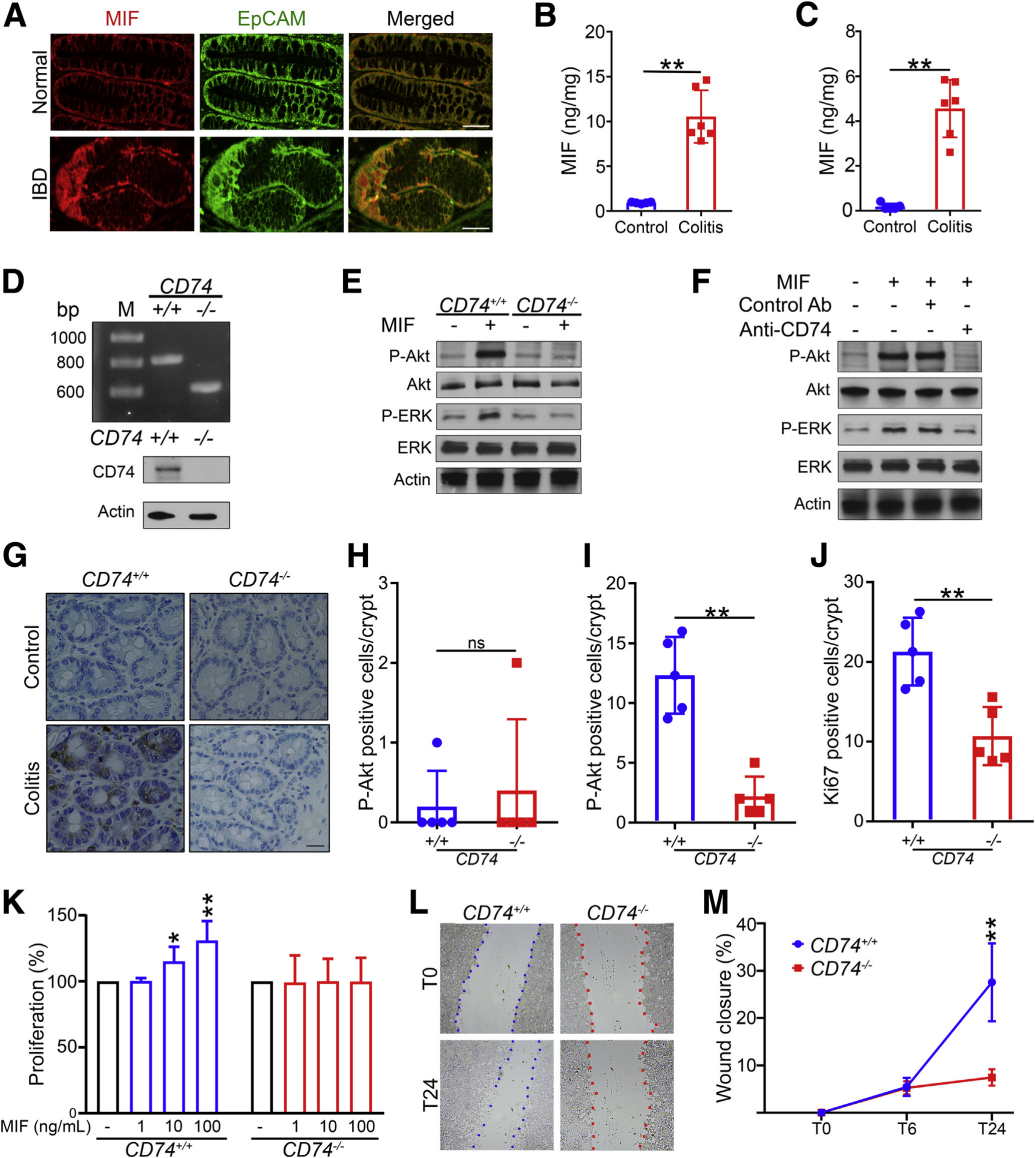
**CD74 signaling activates the pro-proliferative pathways.** (*A*) Confocal immunofluorescence images of intestinal epithelial cells of IBD patient and normal healthy controls expressing MIF. *Scale bars*: 50 μm. (*B* and *C*) Increased MIF secretion in colitis. Intestinal tissue and luminal MIF levels in mice 24 hours after intracecal injection with 3% DSS in drinking water or normal drinking water control (n = 6 per group). (*D*) Genotyping and immunoblot analysis of *CD74*^*+/+*^ and *CD74*^*–/–*^ intestinal epithelial cells. (*E* and *F*) *CD74-*MIF interaction activates Akt and ERK pathways. Immunoblot analysis of Akt and ERK phosphorylation in *CD74*^*+/+*^ and *CD74*^*–/–*^ intestinal epithelial cells stimulated with MIF. Akt and ERK phosphorylation assessed by immunoblot analysis of intestinal epithelial cells stimulated with MIF in the presence of anti-CD74 or control antibodies. Actin served as a loading control. (*G*) Immunohistochemical analysis of Akt phosphorylation in epithelial cells of *CD74*^*+/+*^ and *CD74*^*–/–*^ mice during (*H*) steady-state control and (*I*) colitis conditions (n = 5 per group). (*J*) Intestinal tissue stained for Ki67 by immunohistochemistry and quantitated. *Scale bars*: 50 μm. (*K*–*M*) CD74 stimulation promotes epithelial cell proliferation and wound closure. Percentage proliferation of *CD74*^*+/+*^ and *CD74*^*–/–*^ intestinal epithelial cells 24 hours after dose-dependent stimulation with MIF. Representative images and quantitation of *CD74*^*+/+*^ and *CD74*^*–/–*^ intestinal epithelial cell wound closure at baseline (T0) and after 6 hours (T6) and 24 hours (T24) of MIF (100 ng/mL) stimulation. Data are representative of at least 2 independent experiments. Data represent means and SD. *P < .05,**P < .01. Ab, antibody; EpCAM, epithelial cell adhesion molecule; P-Akt, phosphorylated Akt; P-ERK, phosphorylated ERK.

We investigated the expression of the CD74 ligand MIF in colitis. Epithelial cells constitutively express MIF, with increased secretion during inflammation. Secreted MIF acts in an autocrine or paracrine manner to stimulate cells.^26^ First, we studied the expression MIF in the human colon of patients with IBD and healthy controls. We observed that MIF was strongly expressed in the colonic epithelium as detected by immunofluorescence (Figure 4*A*). Next, we found that MIF levels were increased significantly in the intestinal lumen and tissue of mice during DSS-induced intestinal inflammation in mice (Figure 4*B* and *C*).

We next sought to understand the mechanism by which CD74 promotes gut mucosal repair. CD74 serves as a cell surface receptor for MIF. Cancer biology studies have shown that CD74 bound to MIF initiates survival and cell proliferation through the phosphoinositide-3-kinase (PI3K)/protein kinase B (Akt) and extracellular signal-regulated kinase (ERK) signaling pathways.^27^ In addition, PI3K/Akt and ERK signaling pathways play a crucial role in promoting intestinal epithelial proliferation, survival, and healing.^28^^,^^29^ Therefore, we hypothesized that stimulation of CD74 would result in phosphorylation and activation of Akt and ERK in intestinal cells. Colonic epithelial cells expressing CD74 (*CD74*^*+/+*^) showed enhanced Akt and ERK phosphorylation after stimulation with MIF, which was absent in *CD74*^*−/−*^ cells (Figure 4*D* and *E*). In addition to gene silencing, we used an antibody-mediated neutralization approach to test the effect of CD74 on Akt and ERK activation, finding that MIF-stimulated CD74-dependent Akt, and ERK activation was blocked by specific anti-CD74 antibodies (Figure 4*F*). During inflammation-induced tissue damage, the intestinal epithelium develops hyperproliferative crypts, which are important for re-epithelialization, an essential aspect of mucosal healing.^5^ Because acute intestinal inflammation induces Akt activation,^30^ we also investigated Akt signaling in *CD74*^*–/–*^ mice during DSS-induced intestinal inflammation. We found that there were significantly lower levels of phosphorylated Akt in epithelial crypt cells of *CD74*^*–/–*^ mice compared with WT controls during inflammation (Figure 4*G*–*I*). Consistent with these findings, intestinal epithelial cell proliferation was significantly lower in *CD74*^*–/–*^ mice compared with WT controls during colitis analyzed by Ki67 immunohistochemical staining (Figure 4*J*). Because CD74 stimulation led to Akt and ERK activation, we evaluated the direct effect of CD74 stimulation on intestinal epithelial cells using proliferation and wound healing assays.^31^ We found that MIF stimulation increased in epithelial cell proliferation and wound closure, and this effect was lost in CD74-deficient cells (Figure 4*K*–*M*). Collectively, these findings support that CD74 promotes healing by enhancing epithelial cell regeneration through the activation of Akt and ERK in intestinal epithelial cells.

**Figure 5:**
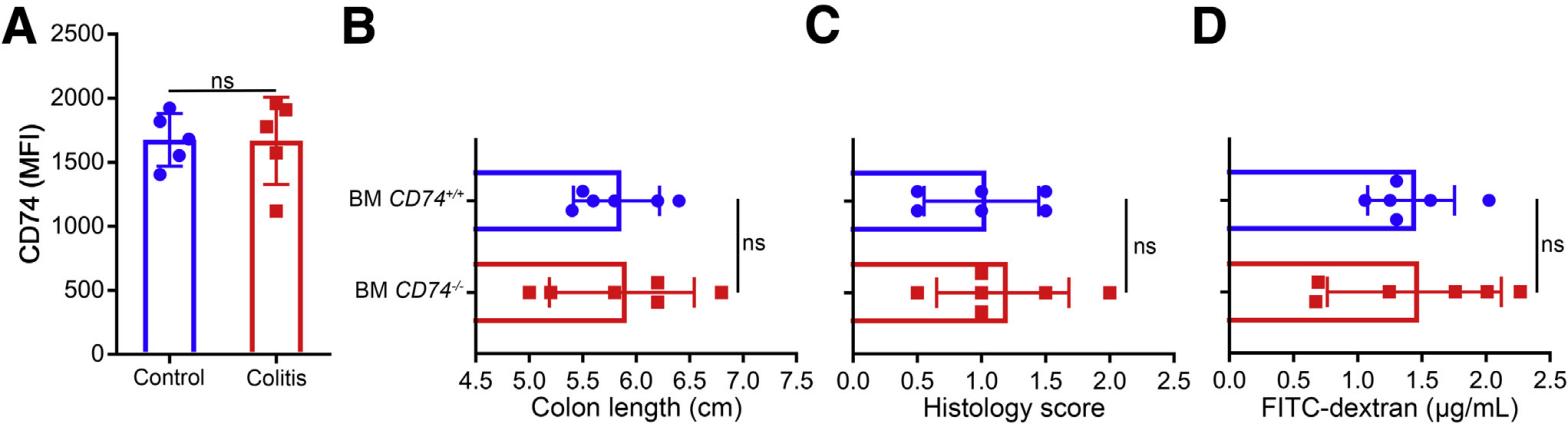
**Role of CD74 signaling in immune cells.** (*A*) Flow cytometry analysis of immune cells for CD74 surface expression after 3% DSS in drinking water or normal drinking water control (n = 5 per group). (*B*) Colon lengths, (*C*) histology scores, and (*D*) fluorescein isothiocyanate (FITC)-dextran levels of mice with *CD74*^*+/+*^ and *CD74*^*–/–*^ bone marrow (BM) treated with DSS followed by a recovery period (n = 6 per group). Data represent means and SD. MFI, mean fluorescent intensity.

We also investigated the possible contribution of immune cells. First, we did not observe a significant change in CD74 expression by immune cells during colitis as analyzed by flow cytometry. Consistent with these findings, we did not observe any significant differences in the healing patterns between WT mice with *CD74*^*+/+*^ or *CD74*^*–/–*^ bone marrow in colitis (Figure 5).

## Discussion

It is crucial that we understand how mucosal healing is regulated because it predicts sustained remission and resection-free survival in patients with IBD.^4^ Injury triggers an inflammatory response that protects against invading pathogens and also activates repair signals that are essential for healing.^6^^,^^7^ However, our current understanding of inflammation-driven repair mechanisms are limited. Here, we studied the role of CD74 signaling in mucosal healing during colitis, finding that CD74 activation does not drive tissue destruction, but protects the host by promoting mechanisms that restore epithelial lining and mucosal integrity during intestinal inflammation.

The renewal of the intestinal epithelium is critical for mucosal healing and is regulated by signaling pathways that control the proliferation of intestinal epithelial cells.^5^^,^^6^^,^^32^, ^33^, ^34^ CD74 signaling was not essential for intestinal homeostasis under steady-state conditions. However, CD74 signaling was strongly activated during the inflammatory response to mucosal injury with overexpression of CD74 most noticeable in the proliferating crypt epithelial cells. PI3K/Akt and ERK signaling are well-characterized pathways that mediate cell proliferation.^35^, ^36^, ^37^, ^38^ In this study, we show that CD74, the receptor for MIF cytokine, triggers activation of Akt and ERK to promote regeneration. Through Akt and ERK, intestinal CD74 signaling enhances epithelial cell proliferation, promoting recovery of mucosal injury. Thus, MIF–CD74–Akt and ERK pathways link inflammation to epithelial cell regeneration, mucosal healing, and repair.

Mucosal healing is essential for proper response to IBD therapy. Anti–tumor necrosis factor (TNF) agents have drastically changed the way to treat IBD, leading to mucosal healing in IBD patients.^39^ Unfortunately, more than half of patients with IBD do not respond to anti-TNF therapy, by mechanisms that are not completely understood. In a recent genetic study, a CD74 polymorphism was associated with anti-TNF treatment failure in patients with IBD,^40^ generating the hypothesis that failure could be more likely to occur in the setting of defective CD74 signaling. It would be interesting to determine whether CD74 gene variants can be used to predict success in a personalized medicine approach to the management of IBD.

New treatments for IBD are needed because many patients do not respond to the clinically approved drugs.^4^^,^^41^ Enhancing or stimulating the CD74 pathway may be a potential therapeutic strategy for facilitating mucosal healing in IBD. Stimulation of CD74 with exogenous MIF might induce mucosal healing. However, administration of MIF has the potential to lead to an excessive inflammatory state. This is because MIF is capable of stimulating receptors other than CD74, such as CXC motif chemokine receptors 2 and 4.^42^^,^^43^ Recent studies have expanded our knowledge of the specific amino acids that enable the interaction between MIF and its receptors, thus, constructing MIF mutant proteins that selectively stimulate CD74 might become possible.^44^ Therefore, new therapies might arise from identifying CD74 agonists that promote the regenerative process while avoiding an exaggerated inflammatory response, which is an area of ongoing research.

In conclusion, we report the observation that CD74 is increased in inflamed tissue in patients with IBD, a disease that affects millions of people worldwide, and provide evidence using human studies, mouse models, and cellular models that CD74 signaling is essential for inflammation-driven repair. These results potentially can be leveraged to create new therapeutic opportunities to address the unmet medical needs for IBD patients.

## Methods

### Human Samples

Intestinal samples from IBD patients and non-IBD healthy controls were obtained from the University of Virginia Biorepository and Tissue Research Facility. Intestinal samples from patients with amebic colitis was provided by the Acquired Immune-Deficiency Syndrome Clinical Center of the National Center for Global Health and Medicine in Japan. Human transcriptomics data were from public data set GSE6731.^45^ Biopsy specimens were taken from healthy persons, and both noninflamed and inflamed colon biopsy specimens from IBD patients (Crohn's disease and ulcerative colitis).

### Mice

*CD74*^*–/–*^ mice in a C57BL/6 background were inbred and generated as previously described.^9^ WT C57BL/6 and C57BL/6 CD45.1 control mice were obtained from Jackson Laboratory (Bar Harbor, ME). *CD74*^*–/–*^ was confirmed via genotyping. Experiments with *CD74*^*–/–*^ mice were performed with co-housed littermate controls.

### DSS and TNBS-Induced Colitis Followed by Recovery Models

Mice received 2.5% (wt/vol) DSS dissolved in drinking water for 7 days followed by normal drinking water for recovery.^32^ Mice were weighed and clinically scored daily, and treatment was discontinued if mice showed greater than 20% weight loss or extreme signs of morbidity.^12^ TNBS injury-repair chronic colitis was induced as described previously.^15^ Mice were euthanized after 5 cycles of TNBS treatment.

### Intestinal Epithelial Cell Isolation

Isolation of intestinal epithelial cells was performed as previously reported.^46^ To induce acute colitis,^32^ 3% DSS was injected intracecally after laparotomy. The entire cecum was collected, and then cut longitudinally to expose the epithelia. The tissue was washed in Hank’s buffered salt solution (HBSS), and placed in 15-mL conicles with 5 mL of epithelial removal buffer (phosphate-buffered saline, 2.5 mmol/L EDTA, 0.75 mmol/L dithiothreitol, and 10 μg/mL DNase I, grade II). The tissue was incubated with rocking at 37°C for 20 minutes. The tubes then were shaken vigorously for 30 seconds while held parallel to the ground to fully separate the epithelium, and then the remnant tissue was removed from the tube. The cells were pelleted and washed with 10 mL phosphate-buffered saline with 10% fetal bovine serum (vol/vol). The cells were incubated with rocking for 10 minutes at 37°C in single-cell buffer (HBSS [Ca2+/Mg2+ free], 1.0 U/mL dispase [Gibco, Gaithersburg, MD], and 10 μg/mL DNase I grade II). The cells were gently passed through a 70-μm filter and washed before staining for flow cytometry.

### Intestinal Lamina Propria Cell Isolation

Lamina propria was prepared for flow cytometry as previously described.^47^ In brief, the cecum was removed, cut longitudinally, and rinsed in HBSS with 5% fetal calf serum and 25 mmol/L HEPES buffer. The tissue was incubated in prewarmed epithelial removal buffer consisting of HBSS, 15 mmol/L HEPES, 5 mmol/L EDTA, 10% fetal calf serum, and 1 mmol/L dithiothreitol at 37°C on a shaking incubator at 220 rpm for 30 minutes. The tissue was thoroughly sliced using scissors and incubated in prewarmed digestion buffer consisting of RPMI containing Liberase TL (Roche, Manheim, Germany) and DNase (Roche) at 37°C on a shaking incubator at 220 rpm for 30 minutes. After digestion, tissue was passed through 100-μm and 40-μm nylon strainers, resuspended in a fluorescence-activated cell sorter buffer, and quantified for total cell numbers and cell viability using trypan blue cell counting before staining for flow cytometry.

### Flow Cytometry

For surface staining, single-cell suspensions were incubated with live/dead stain (Zombie Aqua 405; Biolegend, San Diego, CA), anti-CD74 (LN-2; Santa Cruz, Dallas, TX), anti-CD45 (30-F11; Biolegend), and anti-EpCAM (G8.8; Biolegend) monoclonal antibodies. Samples were run on a FACSCalibur flow cytometer (Becton Dickinson, San Jose, CA) and data were analyzed with FlowJo software (Ashland, OR).

### *E histolytica*–induced colitis

To induce colitis, mice were infected by intracecal inoculation with *E histolytica* trophozoites. A total of 2 × 10^6^ trophozoites in 100 μL of Trypticase yeast extract iron serum 33 media were injected intracecally after laparotomy as previously described.^19^^,^^21^ For antibody-mediated neutralization, 500 μg rat anti-mouse CD74 blocking antibody was injected intraperitoneally 24 hours before and again intracecally at the time of infection.^19^

### Intestinal Permeability and Gut Barrier Function

Intestinal permeability and barrier integrity were assessed using fluorescein isothiocyanate–conjugated dextran as described previously^48^ and by measuring luminal albumin.^32^

### Recombinant Protein Expression, Anti-Mouse CD74 Antibody Purification, and Gene Silencing

Glutathione S-transferase (GST)-CD74, MIF, and GST were expressed and purified as described previously.^49^^,^^50^ Purified GST-CD74 was used to immunize rats (Cocalico Biologicals, Stevens, PA), and pre-immune serum, test bleeds, and the final bleed were received and tested by Western blot. pET28–maltose binding protein (MBP)-Tobacco Etch Virus (TEV) was a gift from Zita Balklava and Thomas Wassmer (plasmid 69929; Addgene, Watertown, MA).^51^ Separately, a MBP fusion of CD74 for purification of CD74 antibody was generated by double digestion of GST-CD74 and pET28-MBP-TEV with BamhI and XhoI followed by transformation of *Escherichia coli* Bl21 competent cells. MBP-CD74 was purified as described previously.^51^ Briefly, MBP-CD74 was affinity-purified with amylose resin (New England Biotechnologies, Ipswich, MA), eluted with 10 mmol/L maltose, and bound to N-hydroxy-succinimide–activated agarose (Pierce, Rockford, IL). Sera was cleared of anti-GST and nonspecific antibodies before affinity purification with MBP-CD74. MBP was expressed from pET28-MBP-TEV and affinity-purified with amylose resin. MBP bound to amylose resin and GST bound to glutathione sepharose 4FF (GE Life Sciences, Pittsburgh, PA) were combined in a single column and mixed with serum overnight. The cleared sera then was collected and incubated overnight with MBP-CD74. Antibodies were eluted in 0.1 mol/L glycine, pH 2.8, and neutralized with 1 mol/L Tris, pH 9.0, and buffer was exchanged into phosphate-buffered saline with a 3-kilodalton MWCO Centrifugal Filter (EMD Millipore, Burlington, MA).^52^ For generation of *CD74*^*–/–*^ cells, pSpCas9(BB)-2A-Puro (PX459) V2.0 was a gift from Feng Zhang (plasmid 62988; Addgene).^53^ pSpCas9(BB)-2A-Puro was digested with BbsI and ligated with 2 sets of CD74 single guide RNA primers: 1 forward: 5’-CACCGAATCTGATTCGTCCACAGA-3’, reverse: 5’-AAACTCTGTGGACGAATCAGATTC-3’; and 2 forward: 5’-CACCGAGAGGTATGTGTGAGCACC-3’, reverse: 5’-AAACGGTGCTCACACATACCTCTC-3’. The ligated Cas9 plasmid then was transformed into E. cloni 5-α chemically competent cells (Lucigen, Middleton, WI). Plasmid then was purified and sequence-confirmed before use for transfection. HCT-116 *CD74*^*–/–*^ cells were generated by incubating cells in FuGENE-single guide RNA complex in Dulbecco’s modified Eagle medium overnight. Transfected cells were selected with 1.0 μg/mL puromycin, and genotyped. HCT116 was obtained from the American Type Culture Collection and tested negative for *Mycoplasma* (Lonza, Walkersville, MD).

### MIF Stimulation, Cell Proliferation, and Wound Healing Assays

MIF stimulation was performed as previously described.^27^ *CD74*^*+/+*^ and CD74^*–/–*^ HCT116 colonic epithelial cells (10^6^/mL) were stimulated with MIF at 100 ng/mL. For antibody neutralization, cells were treated with polyclonal anti-CD74 blocking antibody (Sigma, St. Louis, MO) before MIF stimulation. Cell proliferation and wound closure assays were performed as previously described.^31^

### Enzyme-Linked Immunosorbent Assay

Intestinal tissue was prepared for enzyme-linked immunosorbent assay as described previously.^54^ Intestinal tissue lysates and luminal contents were evaluated by enzyme-linked immunosorbent assay for MIF (R&D Systems, Minneapolis, MN) and albumin (Bethyl Laboratories, Montgomery, TX),^55^ according to the manufacturers’ instructions. Total protein concentration was measured using the Pierce BCA Protein Assay Kit (Thermo Scientific, Rockford, IL).

### Immunohistochemical Staining and Histopathologic Examination

Human and mouse immunohistochemical staining was performed by the University of Virginia Biorepository and Tissue Research Facility.^19^ Staining was performed using the DAKO (Santa Clara, MA) Autostainer Universal Staining System with specific antibodies: sheep anti-mouse CD74 (R&D Systems), rabbit anti-pAkt (S473; Epitomics, Burlingame, CA), rabbit anti-Ki67 (Abcam, Cambridge, MA), and mouse anti-human CD74 (LN-2; Santa Cruz).^56^ Tissue was stained with H&E by the University of Virginia Research Histology Core.^19^ Histologic scoring was performed by 2 independent blinded scorers as previously described.^57^

### Immunofluorescence Staining

Antigen retrieval was completed by the University of Virginia Biorepository and Tissue Research Facility. Mouse tissue was probed with goat anti-EphB2 and anti-EphB3 (R&D Systems),^14^ and sheep anti-CD74 (R&D Systems). Human tissue was probed with rabbit anti-MIF (ThermoFisher, Pittsburgh, PA) and anti-EpCAM (G8.8; eBioscience, Waltham, MA).

### Immunoblotting

Equal cell amounts of HCT116 from both *CD74*^*+/+*^ and *CD74*^*–/–*^ genotypes were lysed with CelLytic M (Sigma) or RIPA (for nuclear proteins), and electrophoresed on 4%–20% TGX gels (Bio-Rad, Hercules, CA). Proteins were transferred to nitrocellulose and blocked with bovine serum albumin. Blots then were probed for proteins with the following antibodies: mouse anti-human CD74 (LN-2; Santa Cruz), rabbit anti-human actin (Sigma), rabbit anti-human pan-Akt (C67E7) and phospho-Akt (Ser473, D9E; Cell Signaling Technologies, Danvers, MA),^27^ rabbit anti-human ERK (137F5) and phospho-ERK (Thr202/Tyr204, D13.14.4E; Cell Signaling Technologies).

### Bone Marrow Chimeras

C57BL/6J mice were irradiated (11 Gy per animal) and reconstituted with bone marrow cells from *CD74*^*–/–*^ or WT mice bearing a congenic marker (CD45.1) as described in similar studies.^34^ Prophylactic antibiotic treatment (trimethoprim/sulfamethoxazole) was provided in drinking water 3 days before and 21 days after radiation. Bone marrow reconstitution was confirmed by flow cytometry 6 weeks after transplant. Eight weeks after transplant, chimera mice were challenged with DSS followed by a recovery period as described earlier.

### Statistics

Statistical differences between 2 groups were determined using the Mann–Whitney *U* and 2-tailed *t* test. Statistical differences between the means of more than 2 groups were analyzed using 1-way analysis of variance with a post hoc test. A *P* value less than .05 was considered statistically significant. Error bars represent means ± SD in all figures.

### Study Approval

All animal procedures were approved by the University of Virginia Institutional Animal Care and Use Committee. All animal studies were performed in compliance with the federal regulations set forth in the Animal Welfare Act, the recommendations in the Guide for the Care and Use of Laboratory Animals of the National Institutes of Health, and the guidelines of the University of Virginia Institutional Animal Care and Use Committee. The retrospective analyses of human colon biopsy material were performed on deidentified material, and thus the studies were exempt from the informed consent requirement.
